# Comparison of the Metastasis Predictive Potential of mRNA and Long Non-Coding RNA Profiling in Systemically Untreated Breast Cancer

**DOI:** 10.3390/cancers13194907

**Published:** 2021-09-29

**Authors:** Thi T. N. Do, Ines Block, Mark Burton, Kristina P. Sørensen, Martin J. Larsen, Martin Bak, Søren Cold, Mads Thomassen, Qihua Tan, Torben A. Kruse

**Affiliations:** 1 Department of Clinical Genetics, Odense University Hospital, 5000 Odense C, Denmark; Nhu.Do@rsyd.dk (T.T.N.D.); Ines.Block@uk-halle.de (I.B.); Mark.Burton@rsyd.dk (M.B.); Kristina.Sorensen1@rsyd.dk (K.P.S.); Martin.Larsen@rsyd.dk (M.J.L.); Mads.Thomassen@rsyd.dk (M.T.); 2 Human Genetics, Department of Clinical Research, University of Southern Denmark, 5000 Odense C, Denmark; Qtan@health.sdu.dk; 3 Clinical Genome Center, University of Southern Denmark & Region of Southern Denmark, 5000 Odense C, Denmark; 4 Department of Pathology, Odense University Hospital, 5000 Odense C, Denmark; Martin.Bak@rsyd.dk; 5 Department of Pathology, Hospital of Southwest Jutland, 6700 Esbjerg, Denmark; 6 Department of Oncology, Odense University Hospital, 5000 Odense C, Denmark; Soeren.Cold@rsyd.dk; 7 Epidemiology, Department of Public Health, University of Southern Denmark, 5000 Odense C, Denmark

**Keywords:** low-risk breast cancer, lymph node negative, systemically untreated patients, long non-coding RNA, mRNA, prognostic predictors, machine learning methods

## Abstract

**Simple Summary:**

To support health care providers in clinical decision-making for breast cancer (BC) patients, profiles of gene activity patterns have previously been developed, where the majority have been based on messenger RNAs (mRNAs), molecules coding for proteins. However, we and others have recently developed profiles based on functional molecules that do not code for proteins—e.g., long non-coding RNAs (lncRNAs)—demonstrating great prognostic potential. Unfortunately, studies comparing such profiles for predicting relapse in BC patients are very scarce. Therefore, we aimed to compare these two types of molecules (mRNAs and lncRNAs) to forecast relapse in low-risk BC patients using advanced machine learning methods with two different approaches. Regardless of approach, our data suggested that profiles based on lncRNAs improved prediction of relapse and demonstrated potential advantages for future profile development.

**Abstract:**

Several gene expression signatures based on mRNAs and a few based on long non-coding RNAs (lncRNAs) have been developed to provide prognostic information beyond clinical evaluation in breast cancer (BC). However, the comparison of such signatures for predicting recurrence is very scarce. Therefore, we compared the prognostic utility of mRNAs and lncRNAs in low-risk BC patients using two different classification strategies. Frozen primary tumor samples from 160 lymph node negative and systemically untreated BC patients were included; 80 developed recurrence—i.e., regional or distant metastasis while 80 remained recurrence-free (mean follow-up of 20.9 years). Patients were pairwise matched for clinicopathological characteristics. Classification based on differential mRNA or lncRNA expression using seven individual machine learning methods and a voting scheme classified patients into risk-subgroups. Classification by the seven methods with a fixed sensitivity of ≥90% resulted in specificities ranging from 16–40% for mRNA and 38–58% for lncRNA, and after voting, specificities of 38% and 60% respectively. Classifier performance based on an alternative classification approach of balanced accuracy optimization also provided higher specificities for lncRNA than mRNA at comparable sensitivities. Thus, our results suggested that classification followed by voting improved prognostic power using lncRNAs compared to mRNAs regardless of classification strategy.

## 1. Introduction

In breast cancer (BC), clinical inter-tumor heterogeneity is represented by staging systems, whereas histopathologic and molecular classification reflect morphologic and genetic inter-tumor heterogeneity [1,2,3]. Intra-tumor heterogeneity also occurs at the genomic, transcriptomic, proteomic, and morphologic level [4,5,6], causing BC patients to possess varying risk for recurrence development [7,8]. For instance, to guide treatment decision, patients are routinely classified based on lymph node status, tumor size, and the positivity or negativity of the tumor for various receptors, such as hormonal receptors or human epidermal growth factor receptor 2 (HER2) (as reviewed by Cardoso et al. [9]).

Due to lack of optimal clinical classification methods, and ability to identify patients with low risk of experiencing recurrence, adjuvant systemic treatments are provided for more than 90% of all current BC patients as they are classified as high-risk. This is despite the fact that up to 40% most likely do not benefit from it, as surgical removal of the primary tumor and radiotherapy often are sufficient to prevent recurrence [10,11]. These patients are thus subjected to unnecessary side effects while increasing the expenditures for the health care system. Research focus has, in the past years, shifted from intensifying treatment to also consider reducing overtreatment, as the BC prognosis due to early diagnosis and advanced treatments has improved over the years. Therefore, it is crucial to predict recurrence at time of diagnosis and determine which patients most likely will never experience recurrence after surgery. By identifying of patients with indolent tumors, we would have the confidence to alleviate the treatments while achieving similar outcomes [12,13,14,15]. 

Since the demonstration of the prognostic role of gene expression profiling in primary breast tumors by van’t Veer et al., several mRNA-based signatures have been developed to provide prognostic information beyond clinical evaluation (e.g., MammaPrint, Oncotype DX, PAM50) [16,17,18]. Despite the focus on mRNAs, gene expression profiles based on long non-coding RNAs (lncRNAs) have recently emerged, demonstrating great prognostic potential in BC [19,20,21]. LncRNAs have become a research area of intensive focus and their biology and roles in tumor development and progression are widely studied. Associations between several lncRNAs and stage, as well as prognosis of multiple tumor types, have been found and the therapeutic potential of lncRNAs has also been extensively studied (as reviewed by Borkiewicz et al. [22]). It is however extremely difficult to generalize findings from such studies across different tumor types due to distinct biology. 

In continuation of this, it is also difficult to generalize biological insights into clinical application. The clinical aspect of lncRNAs as biomarkers for prediction of clinical outcome has been studied to a lesser degree [19,20,21]. Even more limited are studies comparing the relative ability of mRNA and lncRNA-based signatures to predict recurrence. In this study, we compared mRNA and lncRNA-based signatures using two different classification strategies which furthermore were compared for clinical application. This study is, to the best of our knowledge, the first to compare such signatures in systemically untreated low-risk BC patients with very long follow-up.

## 2. Materials and Methods

### 2.1. Tumor Biopsies

The study included frozen tumor biopsies from lymph node negative (LNN) and systemically untreated low-risk BC patients who were diagnosed from 1980 to 2003 on the island of Funen, Denmark. A total of 160 frozen tumor biopsies were collected as previously described [19]. Half of the patients developed recurrence that is, regional or distant metastasis within 10 years after diagnosis while the remaining half were recurrence-free (mean follow-up of 20.9 years). Patient biopsies were pairwise matched according to age (range: 33–88 years), tumor type, tumor diameter (range: 4–50 mm), year of surgery, receptor status (ER, PR, n/a), and histological grade (grade 1–3 or n/a) (Table 1). 

Independence of traditional prognostic markers was achieved by pairwise matching the patients, increasing the power by enriching informative clinical endpoints while bias related to sample retrieval, storage, and diagnostic procedures was reduced. All clinicopathological information used for sample matching was acquired from the Danish Breast Cancer Cooperative Group (DBCG) database, the Funen pathology database, or the nationwide pathology database. The study was approved by the Danish National Committee on Health Research (S-VF-20020142). The study was retrospective, and no informed consent was obtained from the included patients as approved by the Ethical Committee [19].

### 2.2. Microarray Analysis and Re-Annotation

Total RNA was extracted from freshly frozen tumor biopsies as previously described [23]. A modified standard design of the SurePrint G3 Human GE 8 × 60k oligonucleotide slides (G4102A) by Agilent Technologies (Santa Clara, Santa Clara County, CA, USA) was applied for gene expression analysis. The matched sample pairs were kept together during all steps of RNA extraction, hybridization, and gene expression analysis. LncRNA and mRNA expression data have previously been deposited in NCBI’s Gene Expression Omnibus [24] and are accessible through GEO Series accession number GSE48408 [19]. To select the probes covering mRNAs and lncRNAs, chromosomal positions of the probes in the annotation file from Agilent were matched to the positions in the Human RNA catalog from GENCODE v. 16 [25] as previously described [19,26].

Gene expression data were analyzed in 160 freshly frozen primary tumors from LNN and systemically untreated low-risk patients with invasive BC. Re-annotation resulted in identification of 21,858 and 4810 mRNA and lncRNA probes on the Agilent array. Further analysis was performed using all mRNA probes and probes covering 2811 unique lncRNAs (26). Comparing the two patient groups, recurrence development vs. recurrence-free using paired *t*-tests identified 148 and 160 differentially expressed lncRNAs and mRNAs (FDR ≤ 0.05). The lists of the differentially expressed RNAs are shown in Tables S1 and S2. 

To avoid any bias, the input lists of RNA transcripts for the procedure of outcome prediction using leave-one-pair-out cross-validation (LOPOCV) were applied unmodified, i.e., with 21,858 and 2811 mRNAs and lncRNA probes. 

### 2.3. Data Processing, Classification and Voting

**Figure A1:**
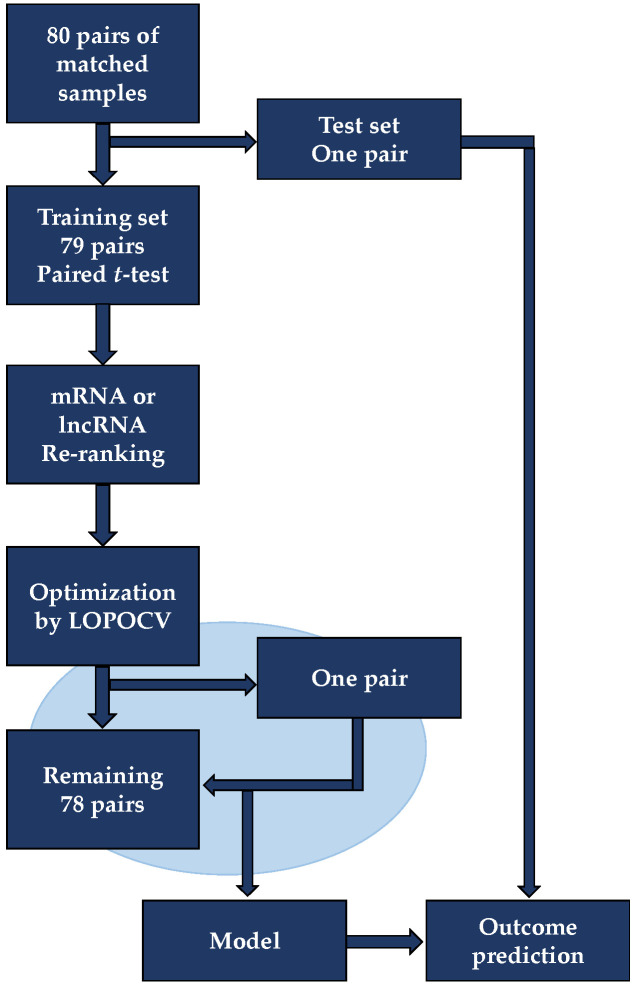
Outcome prediction using leave-one-pair-out cross-validation. In this procedure, a single pair of matched samples served as test samples while the remaining 79 sample pairs served as a training set. For feature selection in the training set, Student’s two-tailed *t*-test was used to identify differentially expressed mRNAs and lncRNAs (FDR ≤ 0.05) where these subsequently were re-ranked according to their random forest-based variable importance value. Using leave-one-out-cross-validation (LOPOCV), each of the seven machine learning methods identified the optimal mRNA and lncRNA set by using the highest ranked mRNAs/ lncRNAs in a top-down forward-wrapper approach where outcome in one selected pair was predicted using the remaining 78 pairs as training set (inner loop, blue). The inner loop procedure was repeated until each pair had been left out once. The final optimal mRNA or lncRNA set was used to predict outcome in the initially left out test pair. The entire procedure was repeated until all pairs had been left out as a test pair and their outcome independently predicted by the inner training loop.

Array scanning and data preprocessing were performed as previously described [23]. Seven of the most commonly used machine learning methods, found through literature search on breast cancer classification were applied for sample classification: linear discriminant analysis (LDA), support vector machines based on a radial kernel (R-SVM) or linear kernel (L-SVM), random forest (RF), naïve Bayes (NB), COX risk score (COX-RS), and logistic regression (LR). LOPOCV was applied to provide an unbiased estimate of classifier performance (Figure A1). This approach has proven to be optimal for analysis of smaller datasets [27,28]. We applied a voting scheme for gene expression-based classification of BC patients, which previously has been shown to improve classification performance [29]. 

**Figure 1:**
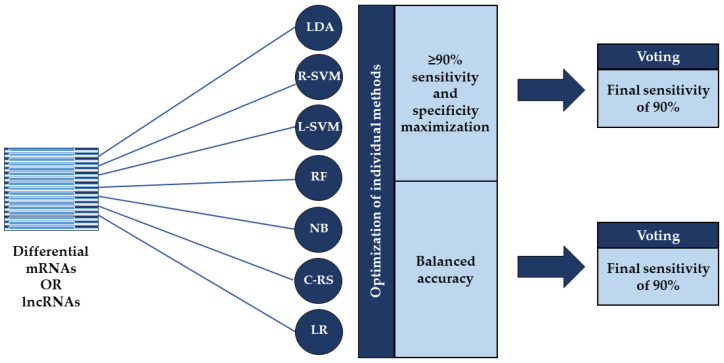
Overview of the two applied classification strategies. Classification was performed according to the differentially expressed mRNAs or lncRNAs using seven machine learning methods: linear discriminant analysis (LDA), support vector machines based on a radial kernel (R-SVM) or linear kernel (L-SVM), random forest (RF), naïve Bayes (NB), COX risk score (C-RS), and logistic regression (LR). The individual methods were optimized using two different approaches: one that provided ≥90% sensitivity while maximizing specificity and one that optimized balanced accuracy where both were followed by voting with a final sensitivity of 90%.

All classification procedures and statistical analyses were performed using the R open-source environment (version 4.0.2, https://cran.r-project.org/, accessed on 23 June 2020). For SVM and NB based classification, we used the *e1071* R-package (https://cran.r-project.org/web/packages/e1071/index.html, accessed on 23 June 2020 for *e1071* and the following R-packages), while the RF, COX-RS, LDA, and LR based classification procedures were performed using the *randomForest* (https://cran.r-project.org/package=randomForest, accessed on 23 June 2020), *survival* (https://cran.r-project.org/package=survival, accessed on 23 June 2020), *MASS* (https://cran.r-project.org/package=MASS, accessed on 23 June 2020), and *stats (*https://stat.ethz.ch/R-manual/R-devel/library/stats/html/stats-package.html, accessed on 23 June 2020) R-packages, respectively. We performed classification according to the differentially expressed RNA molecules with above mentioned machine learning methods, applying two different approaches which: (1) provided ≥90% sensitivity while maximizing specificity; and (2) optimized balanced accuracy (bAcc). Both optimization strategies were followed by voting with a final sensitivity of 90% (Figure 1). Balanced accuracy is the arithmetic mean of two metrics: sensitivity, which is the proportion of truly affected subjects who are correctly classified as positive; and specificity, which measures the proportion of truly unaffected subjects who are correctly identified as negative [30]. 

The integrated voting results were obtained using seven distinctive cutoffs ranging from 1–7, representing different degrees of classification agreement between the seven methods in terms of recurrence votes. A cutoff of 1 meant that one or more votes for recurrence, placed the patient as high-risk whereas zero votes classified the patient as low-risk, while a cutoff of 7 meant that if all votes were assigned for recurrence, the patient was placed as high-risk, while six or less votes corresponded to low-risk.

A more detailed description of the applied procedures is provided in the Appendix A.

### 2.4. Statistical Analysis

Paired Student’s two-tailed *t*-tests were performed for analysis of differential mRNA and lncRNA expression where a false discovery rate (FDR) ≤0.05 was considered significant. Furthermore, a one-sided two-proportion z-test was used to compare the estimated significance between each of the seven methods using lncRNA or mRNA as explanatory variables (H_A_: lncRNA performance > mRNA performance) by application of the *prop.test* function embedded in the *stats* R-package. 

## 3. Results

Gene expression data were analyzed in 160 freshly frozen primary tumors from LNN and systemically untreated low-risk patients with invasive BC. Re-annotation resulted in identification of 21,858 and 4810 mRNA and lncRNA probes on the Agilent array. Further analysis was performed using all mRNA probes and probes covering 2811 unique lncRNAs [26]. Comparing the two patient groups, recurrence development vs. recurrence-free using paired *t*-tests identified 148 and 160 differentially expressed lncRNAs and mRNAs (FDR ≤ 0.05).

### 3.1. Classification with ≥90% Sensitivity Threshold Followed by Voting

A cumulative risk of recurrence of ≥10% within 10 years is defined as high-risk and in Denmark adjuvant systemic therapy is offered to patients with high risk of recurrence [31]. Classification was therefore conducted with a threshold that provided ≥90% sensitivity to identify patients eligible for systemic treatment. Applying this criterion for the seven methods, classification based on mRNA or lncRNA divided our patients into subgroups with high or low risk of recurrence. 

An overall classification accuracy for mRNA data ranged from 53% (90% sensitivity, 16% specificity) using LR to 66% (91% sensitivity, 40% specificity) using RF. For lncRNA-based classification, the overall accuracy ranged from 64% (91% sensitivity, 38% specificity) using both the NB and COX-RS methods to 74% (90% sensitivity, 58% specificity) using LDA (Table 2). The specificities obtained by the seven classification methods were consistently higher when using lncRNA compared to mRNA. The classification results with associated *p*-values are shown in Table 2.

In the clinically most relevant classification scheme, both mRNA and lncRNA-based voting obtained a sensitivity of 91% at a voting cutoff of 5 where the corresponding specificity was 38% when mRNA was used and 60% for lncRNA. The difference between lncRNA and mRNA performance was significant at a *p*-value of 0.013 (Table 3). The individual voting decisions are summarized in Tables S3–S6. 

### 3.2. Classification with Balanced Accuracy Optimization Followed by Voting

Using optimized bAcc for class assignment by each machine learning method provided an overall classification accuracy for mRNA data ranging from 64% (69%, 68%, 65% sensitivity; 60%, 60%, 64% specificity) using COX-RS, LDA, and LR to 70% (75% sensitivity, 65% specificity) using the NB method. For lncRNA-based classification, the accuracy ranged from 70% (69% sensitivity, 71% specificity) using NB to 78% (79% sensitivity, 76% specificity) using the LR method (Table 4). For six of out seven machine learning methods, the bAcc were higher when classification was based on lncRNA compared to mRNA, while using NB provided equal performance. The classification results with associated p-values are shown in Table 4.

For the integrated voting results, a sensitivity of 92% was obtained at a voting cutoff of 1 when mRNA was applied where the corresponding specificity was 29%. At the same cutoff, lncRNA-based voting resulted in a sensitivity of 88% and specificity of 51%. The voting results with associated p-values are shown in Table 5.

### 3.3. Classification of ER Positive Breast Cancer Patients Followed by Voting 

To make our patient group more homogeneous, we considered merely concordant pairs of estrogen receptor (ER) positive patients, retaining a total of 55 patient pairs. Eight hundred and thirty-six mRNAs and 208 lncRNAs were significantly differentially expressed among the ER positive patients (FDR ≤ 0.05). Classification of ER positive BC patients conducted with ≥90% sensitivity, resulted in an overall classification accuracy for mRNA data ranging from 51–68% whereas the accuracy for lncRNA-based classification ranged from 55–68% (Table A1). Voting based on mRNA obtained a sensitivity of 96% at a voting cutoff of 5 where the corresponding specificity was 38%. At the same cutoff, lncRNA-based voting resulted in a sensitivity of 95% with specificity of 44%. The voting results with associated *p*-values are shown in Table A2. 

Classification conducted with optimized bAcc for class assignment resulted in an overall classification accuracy for mRNA data ranging from 62–70%, whereas the accuracy for lncRNA-based classification ranged from 60–69% (Table A3). Voting using mRNA data obtained a sensitivity of 85% at a cutoff of 1 where the corresponding specificity was 45%. LncRNA-based voting at the same cutoff resulted in a sensitivity of 91% with specificity of 36%. The voting results with associated p-values are shown in Table A4.

## 4. Discussion

Our primary objective was to compare the relative ability of mRNA and lncRNA-based signatures to predict recurrence in systemically untreated BC patients using two different classification strategies (Figure 1), which additionally were compared for clinical application. Microarray gene expression analysis identified differentially expressed mRNAs and lncRNAs between matched patient pairs with and without recurrence development where classification subsequently was performed according to these. As the samples were hybridized simultaneously on the same platform, it markedly reduced inter-platform differences when evaluating differential RNA expression and thus strengthened the results. 

### 4.1. Classification Using lncRNA Compared to mRNA Improved Prognostic Power

For both mRNA and lncRNA data, and regardless of classification strategy, each of the seven machine learning methods achieved an overall classification accuracy of >50%, indicating an above random distinction between recurrence developing and recurrence-free patients (Table 2 and Table 4). Classification conducted with ≥90% sensitivity, resulted in an overall accuracy for mRNA ranging from 53–66%, whereas the accuracies using lncRNA were noticeably higher, ranging from 64–74% (Table 2). The same tendency was observed using classification with bAcc optimization, where accuracies for mRNA ranged from 64–70% and 70–78% for lncRNA (Table 4). Thus, applying either classification strategy supported the same finding: using the seven machine learning methods, lncRNA compared to mRNA-based signatures improved prognostic accuracy.

For decades, mRNA turnover has been a subject of intensive research and with the characterization of lncRNAs, some similarities and differences between their turnovers have been described. As mature lncRNAs like mRNAs are modified with 5′ caps and 3′-poly adenosine (poly(A)) tails, many mechanisms involved in post-transcriptional mRNA decay are believed to also modulate lncRNA degradation [32]. Other similarities include RNA-binding proteins (RBPs) and microRNAs which have been shown to drive the turnovers of both RNA molecules [33,34,35,36]. However, unlike mRNAs, the majority of lncRNAs are not translated into proteins and therefore it is likely that degradation processes associated with the translation machinery are different, leading to individual rates of turnover [32]. Interestingly, some lncRNAs have furthermore been found to form conserved triple-helix complexes which protect them from exonucleases, averting rapid degradation [37,38]. The rate of transcription and degradation of mRNAs and lncRNAs ultimately enables their immense impact on gene regulation and their distinct turnover rates might be reflected in our findings of lncRNAs as seemingly better prognostic predictors compared to mRNAs. However, many additional aspects and features of lncRNA turnover still await exploration and characterization by future studies.

### 4.2. Classification Followed by Voting Supports lncRNAs as Better Prognostic Predictors

To improve classification performance, we applied a voting scheme that allowed the seven methods to vote on whether a sample belonged to the patient group with or without recurrence development. mRNA-based accuracies derived from the integrated voting results following classification with ≥90% sensitivity threshold, ranged from 50–64%, whereas higher accuracies of 58–78% were obtained for lncRNA (Table 3). The integrated voting results after classification using the alternative strategy of bAcc optimization also supported lncRNAs as better prognostic predictors with accuracies ranging from 69–76% for lncRNA while mRNA-based voting obtained 61–70% (Table 5). 

The number of studies comparing the relative ability of mRNA and lncRNA-based signatures to predict recurrence are very limited. However, a study by Xu et al., aimed to identify biomarkers that could improve prognostic predictions [39]. By comparing the expression of lncRNA, mRNA, microRNA, and DNA methylation, they found lncRNAs to be the best prognostic predictors in validated cohorts of four cancer types including BC [39], supporting our findings. However, of the breast tumors included in their study (with comprehensive clinicopathological information), more than half were lymph node positive. Furthermore, they had a short median overall follow-up of 17 months and a small fraction of overall survival events (93 out of 818 patients). To the best of our knowledge, no other studies have performed these comparisons in LNN and systemically untreated BC patients with very long follow-up. 

### 4.3. Comparison of the Two Voting Strategies for Clinical Signature Development 

If we could improve prediction of recurrence at time of diagnosis, a more accurate prognosis could be established for low-risk BC patients and we would thereby achieve substantial reduction of adjuvant systemic therapy application [12,13,14,15]. To elucidate and compare clinical application of the two classification strategies for future signature development, we considered the amount of correctly classified patients in the two groups. We would consistently spare more recurrence-free patients unnecessary adjuvant systemic therapy using lncRNA compared to mRNA-based voting following classification with ≥90% sensitivity. Applying this threshold for lncRNA-based voting with a cutoff of 5, we correctly spare 48 out of 80 (60%) recurrence-free patients adjuvant systemic therapy while correctly classifying 73 out of 80 (91%) patients with recurrence development. Compared to mRNA-based voting, it is 18 additional patients that possibly could have been taken off treatment today (60% vs. 38% specificity) while maintaining a similar sensitivity of 91% (*p*-value of 0.0013, Table 3). 

Making the strategy of optimized bAcc for lncRNA-based voting equally relevant in terms of clinical application, thus, applying a cutoff of 1, we correctly spare 41 out of 80 (51%) recurrence-free patients unnecessary therapy while correctly classifying 70 out of 80 (88%) recurrence developing patients. Compared to mRNA-based voting, it is similarly 18 additional patients which possibly could have been taken off treatment today (51% vs. 29% specificity), although at a slight cost of sensitivity (88% vs. 92% sensitivity, *p*-value of 0.083, Table 5). 

In a clinical situation, the goal would be a final sensitivity after voting of close to 90%, i.e., nearly all patients who develop metastases need to be classified as high-risk. For which of the two initial classification schemes in the individual machine learning methods: (1) Optimization of specificity with sensitivity set at 90% (Table 2); or (2) Optimization of bAcc (Table 4), can we obtain the highest final specificity (correct classification of patients as low-risk)? By comparing results shown in Table 3 and Table 5, it can be seen that highest specificity (with final sensitivity fixed at 90%) can be obtained when the sensitivity was fixed at 90% in the seven initial classifiers.

### 4.4. Comparison of the Two Voting Strategies for Clinical Application in ER Positive Patients

For ER positive BC patients, classification followed by voting conducted with ≥90% sensitivity could likewise spare more recurrence-free patients unnecessary adjuvant systemic therapy using lncRNA compared to mRNA while achieving almost similar sensitivity (Table A2). As our patient group became more homogeneous and as the classification models were developed in a majority of ER positive samples, we could expect this finding. The increased sample homogeneity was also reflected in identification of a larger amount of differentially expressed RNA molecules in ER positive patients. 

The potential advantages of lncRNAs compared to mRNAs was however not as distinctive when applying the alternative strategy of bAcc optimization, indicating that the fewer ER negative samples contributed to a more heterogeneous data structure, leading to better distinction between the two patient groups when using lncRNA data compared to mRNA. LncRNA-based voting was still favored for clinical application when using this strategy as it, unlike mRNA, enabled a sensitivity of ≥90% (Table A4). 

### 4.5. LncRNAs and Their Countless Roles

Although we have more than seven times as many unique mRNA than lncRNA probes on the array (21,858 vs. 2811), paired Student’s two-tailed *t*-tests identified almost the same number of differentially expressed mRNAs and lncRNAs (160 and 148) and thus, a much higher percentage of lncRNAs were found to be statistically significant. Considering this, along with the mentioned findings of lncRNAs as seemingly better prognostic predictors of recurrence in low-risk BC patients, lncRNAs are suggested to be far more informative than mRNAs, indicating that they must have crucial roles in BC progression. 

Several links between lncRNAs and cancer are now known where alteration of lncRNA expression in cancer cells have been demonstrated in multiple cancers besides BC, e.g., prostate and non-small cell lung cancer [23,40,41,42,43]. It is however very difficult to generalize findings across different cancer types in different clinical settings. 

The roles of lncRNAs are vast and intricate as they interact with DNA elements, mRNAs, microRNAs, and proteins where their multifunctional regulatory roles are exerted on multiple levels: epigenetic, peptide-mediated, transcriptional, and post-transcriptional [44]. The diversity of their regulatory roles also includes crucial functions throughout the steps of the metastatic cascade. Different lncRNAs—e.g., H19 and PTAR—have been described in the step of epithelial-mesenchymal transition whereas others—e.g., NORAD and MALAT1—are involved in the promotion of invasion and colonization, respectively (as reviewed by Liu et al. [45]). Exciting lncRNA discoveries hold for future studies where a profound comprehension of the functional roles and mechanisms of lncRNAs along with their complex regulatory network will be explored. 

## 5. Conclusions

The majority of gene expression signatures providing prognostic information in BC have primarily been developed on mRNAs with a few on lncRNAs. The lack of studies comparing such signatures in low-risk BC patients make this study highly relevant. 

In the clinically most relevant classification scheme both mRNA and lncRNA-based voting obtained a sensitivity of 91% at a voting cutoff of 5 where the corresponding specificity was 38% when mRNA was used and 60% for lncRNA. The difference between lncRNA and mRNA performance was significant at a *p*-value of 0.013 (Table 3).

Classification with a fixed sensitivity of ≥90% for the individual machine learning methods followed by voting with a final sensitivity of 90%, obtained consistently higher overall accuracies when based on lncRNAs compared to mRNAs. Similar findings were observed using the alternative strategy of bAcc optimization and thus, classification followed by voting suggested improved prognostic power using lncRNAs compared to mRNAs. Comparing the two classification strategies for clinical application, suggested that development of future RNA-based signatures for assisting clinical decision-making, could gain prognostic power using lncRNA-based classification with ≥90% sensitivity followed by voting with a final sensitivity of 90%. 

In summary, our data suggest that in a group of BC patients, lncRNAs are more informative than mRNAs in prediction of recurrence. We additionally propose a favorable optimizing and classification strategy and thus, we hope that these data encourage other research groups to at least include lncRNAs for their signature development in low-risk BC.

## Figures and Tables

**Table 1:** Patient and tumor characteristics.

Characteristics	Recurrence Development	Recurrence-Free
No. of patients	80 (50)	80 (50)
Age at diagnosis (range: 33–88 years) ≤50 years >50 years	14 (8.8) 66 (41.3)	10 (6.3) 70 (43.8)
Tumor size		
<2 cm	27 (16.9)	30 (18.8)
2–5 cm	53 (33.1)	49 (30.6)
n/a		1 (0.6)
Estrogen receptor status ^a^		
Positive	52 (32.5)	50 (31.3)
Negative	22 (13.8)	24 (15)
n/a	6 (3.8)	6 (3.75)
Tumor type		
Invasive ductal carcinoma (IDC)	62 (38.8)	65 (40.6)
Invasive lobular carcinoma (ILC)	9 (5.6)	9 (5.6)
Mucinous carcinoma	2 (1.3)	2 (1.3)
Papillary carcinoma	3 (1.9)	2 (1.3)
Carcinoma with metaplasia	2 (1.3)	2 (1.3)
n/a	2 (1.3)	-
Histologic grade		
1 (good)	12 (7.5)	15 (9.4)
2 (intermediate)	28 (17.5)	25 (15.6)
3 (poor)	22 (13.8)	24 (15)
n/a	18 (11.3)	16 (10)
Median year of surgery (range 1980–2003)	1993	1994
Mean time to recurrence (months)	58.5	n/a
Mean follow-up (months)	88.3	250.35
Alive at end of follow-up	1	48

n/a: not available/applicable; ^a^ as defined by immunohistochemistry. Values in parentheses indicate percentage of patients in each category out of the whole sample size.

**Table 2:** Classification with ≥90% sensitivity threshold using seven individual machine learning methods.

mRNA				lncRNA			
Method	Sensitivity	Specificity	Accuracy ^a^	Sensitivity	Specificity	Accuracy ^a^	*p* ^b^
LDA	90	30	60	90	58	74	0.0055
R-SVM	90	32	61	90	53	71	0.038
L-SVM	91	32	62	90	50	70	0.082
RF	91	40	66	90	52	71	0.20
NB	91	31	61	91	38	64	0.33
COX-RS	90	21	56	91	38	64	0.089
LR	90	16	53	90	50	70	0.0013

Classification was conducted with a threshold that provided at least 90% sensitivity while maximizing specificity and performances assessed by leave-one-pair-out cross-validation using linear discriminant analysis (LDA), support vector machines based on a radial kernel (R-SVM) or linear kernel (L-SVM), random forest (RF), naïve Bayes (NB), COX risk score (COX-RS), and logistic regression (LR). ^a^ Mean of sensitivity and specificity. All measures are specified in percent. ^b^ *p*-value determined by a one-sided two-proportion z-test comparing the estimated significance between the seven machine learning methods using mRNA or lncRNA.

**Table 3:** Integrated voting results following classification with ≥90% sensitivity threshold.

mRNA				lncRNA			
No. of Rec. Votes	Sensitivity	Specificity	Accuracy ^a^	Sensitivity	Specificity	Accuracy ^a^	*p* ^b^
≥1	100	0	50	98	19	58	0.093
≥2	100	10	55	92	29	61	0.17
≥3	99	20	59	92	41	67	0.086
≥4	96	28	62	92	50	71	0.056
≥5	91	38	64	91	60	76	0.013
≥6	83	45	64	88	64	76	0.013
7	65	64	64	80	75	78	0.0042

The voting results were obtained using seven distinctive cutoffs ranging from 1–7, representing different degrees of classification agreement between the seven machine learning methods in terms of recurrence (rec.) votes. A cutoff of 1 meant that one or more votes for recurrence, placed the patient as high-risk whereas zero votes classified the patient as low-risk and likewise for the rest of the cutoffs. ^a^ Mean of sensitivity and specificity. All measures are specified in percent. ^b^ *p*-value determined by a one-sided two-proportion *z*-test comparing the estimated significance between the seven machine learning methods using mRNA or lncRNA.

**Table 4:** Classification with optimized balanced accuracy using seven individual machine learning methods.

mRNA				lncRNA			
Method	Sensitivity	Specificity	Accuracy ^a^	Sensitivity	Specificity	Accuracy ^a^	*p* ^b^
LDA	68	60	64	75	74	74	0.035
R-SVM	70	66	68	76	68	72	0.26
L-SVM	72	61	67	72	71	72	0.20
RF	71	65	68	75	71	73	0.20
NB	75	65	70	69	71	70	0.50
COX-RS	69	60	64	68	74	71	0.11
LR	65	64	64	79	76	78	0.0042

Classification was conducted with optimized balanced accuracy and performances assessed by leave-one-pair-out cross-validation using linear discriminant analysis (LDA), support vector machines based on a radial kernel (R-SVM) or linear kernel (L-SVM), random forest (RF), naïve Bayes (NB), COX risk score (COX-RS), and logistic regression (LR). ^a^ Mean of sensitivity and specificity. All measures are indicated in percent. ^b^ *p*-value determined by a one-sided two-proportion z-test comparing the estimated significance between the seven machine learning methods using mRNA or lncRNA.

**Table 5:** Integrated voting results following classification using balanced accuracy optimization.

mRNA				lncRNA			
No. of Rec. Votes	Sensitivity	Specificity	Accuracy ^a^	Sensitivity	Specificity	Accuracy ^a^	*p* ^b^
**≥1**	92	29	61	88	51	69	0.083
**≥2**	82	46	64	84	59	71	0.11
**≥3**	78	62	70	80	65	73	0.32
**≥4**	69	66	68	75	72	74	0.14
**≥5**	62	74	68	70	76	73	0.20
**≥6**	53	85	69	65	88	76	0.10
**7**	38	95	66	53	94	73	0.11

The voting results were obtained using seven distinctive cutoffs ranging from 1–7, representing different degrees of classification agreement between the seven machine learning methods in terms of recurrence (rec.) votes. A cutoff of 1 meant that one or more votes for recurrence, assigned the patient to the high-risk group whereas zero votes classified the patient as low-risk and similarly for the rest of the cutoffs. ^a^ Mean of sensitivity and specificity. All measures are indicated in percent. ^b^ *p*-value determined by a one-sided two-proportion z-test comparing the estimated significance between the seven machine learning methods using mRNA or lncRNA.

## Data Availability

The data presented in this study are openly available in the National Center of Biotechnology Information’s Gene Expression Omnibus [24] and are accessible through GEO Series accession number GSE48408 [19].

## Supplementary Materials

The following are available online at https://www.mdpi.com/article/10.3390/cancers13194907/s1, Table S1: Significant mRNAs. Table S2: Annotated significant lncRNAs. Table S3: mRNA-based classification decisions. Table S4: LncRNA-based classification decisions. Table S5: Classification decisions based on mRNAs and lncRNAs, sorted by lncRNAs. Table S6: Classification decisions based on mRNAs and lncRNAs, sorted by mRNAs.

## Appendix A

### Appendix Methods

#### Classification

We performed classification according to the differentially expressed RNA molecules using seven machine learning methods and applying two different approaches in which the first provided ≥90% sensitivity while maximizing specificity whereas the other optimized balanced accuracy. Both strategies were followed by voting with a final sensitivity of 90%. For the first approach, the optimal model during training was selected based on a fixed sensitivity of ≥90% while the specificity was maximized, assessed by leave-one-pair-out cross-validation (LOPOCV). Using the second approach, the optimal model was determined based on mean sensitivity and specificity maximization, likewise assessed by LOPOCV.

In brief, LOPOCV is a procedure that evaluates how well a machine learning method performs by using a single pair of matched samples as test samples, while the remaining samples serve as a training set. Each single pair of matched samples was thus tested individually, and the procedure was repeated until all pairs of matched samples had been left out once. The accuracy of the machine learning methods was determined by the amount of correctly classified samples. An unbiased performance estimate is provided by this procedure, and this approach has furthermore proven to be optimal for analysis of smaller datasets [27,28].

Feature selection is required in the training set to avoid a small sample-per-feature ratio and it has been shown to provide better classification [28]. In this study, the feature selection process comprised of three steps: 1) Testing the mRNAs and lncRNAs in the training set for significant differential expression using paired Student’s two-tailed *t*-test (FDR ≤ 0.05); 2) Ranking the differentially expressed mRNAs and lncRNAs according to their random-forest importance value which describes the standardized drop in prediction accuracy when the class levels are permuted [46]; 3) By subsequently adding one mRNA or lncRNA at a time in a top-down forward-wrapper approach starting with top two mRNAs or lncRNAs from the ranked list followed by classification accuracy assessment. The optimal number of mRNAs and lncRNAs were determined by each of the seven machine learning methods. At each addition, the classification accuracy of the training samples was evaluated using LOPOCV in a nested inner loop [29]. To calculate the significance of the classification results, a one-sided two-proportion z-test was applied, comparing the estimated significance between the seven machine learning methods using mRNA or lncRNA. The pipeline for these classification steps is shown in Figure A1.

**Table A1:** Classification of ER positive BC patients with ≥90% sensitivity threshold using seven individual machine learning methods.

mRNA				lncRNA			
Method	Sensitivity	Specificity	Accuracy ^a^	Sensitivity	Specificity	Accuracy ^a^	*p* ^b^
LDA	98	16	57	95	31	63	0.22
R-SVM	96	40	68	91	35	63	0.74
L-SVM	91	42	66	91	38	65	0.51
RF	91	31	61	93	44	68	0.17
NB	91	35	63	93	36	65	0.43
COX-RS	91	11	51	91	35	63	0.048
LR	91	15	53	93	16	55	0.44

Classification was conducted with a threshold that provided at least 90% sensitivity while maximizing specificity and performances assessed by leave-one-pair-out cross-validation using linear discriminant analysis (LDA), support vector machines based on a radial kernel (R-SVM) or linear kernel (L-SVM), random forest (RF), naïve Bayes (NB), COX risk score (COX-RS), and logistic regression (LR). ^a^ Mean of sensitivity and specificity. All measures are indicated in percent. ^b^ *p*-value determined by a one-sided two-proportion z-test comparing the estimated significance between the seven machine learning methods using mRNA or lncRNA.

**Table A2:** Integrated voting results for ER positive BC patients following classification with ≥90% sensitivity threshold.

mRNA				lncRNA			
No. of Rec. Votes	Sensitivity	Specificity	Accuracy ^a^	Sensitivity	Specificity	Accuracy ^a^	*p* ^b^
≥1	100	0	50	100	2	51	0.49
≥2	100	2	51	100	9	55	0.32
≥3	98	13	55	98	18	58	0.38
≥4	98	18	58	98	33	65	0.18
≥5	96	38	67	95	44	69	0.43
≥6	87	49	68	87	60	74	0.20
7	69	69	69	71	73	72	0.37

Voting was conducted using seven distinctive cutoffs ranging from 1–7, representing different degrees of classification agreement between the seven machine learning methods in terms of recurrence (rec.) votes. A cutoff of 1 meant that one or more votes for recurrence, allocated the patient to the high-risk group whereas zero votes classified the patient as low-risk and likewise for the rest of the cutoffs. ^a^ Mean of sensitivity and specificity. All measures are indicated in percent. ^b^ *p*-value determined by a one-sided two-proportion z-test comparing the estimated significance between the seven machine learning methods using mRNA or lncRNA.

**Table A3:** Classification of ER positive BC patients with optimized balanced accuracy using seven individual classifiers.

mRNA				lncRNA			
Method	Sensitivity	Specificity	Accuracy ^a^	Sensitivity	Specificity	Accuracy ^a^	*p* ^b^
LDA	62	62	62	56	65	61	0.51
R-SVM	75	65	70	69	69	69	0.51
L-SVM	67	71	69	60	73	66	0.63
RF	67	64	65	65	67	66	0.49
NB	62	76	69	62	71	66	0.63
COX-RS	62	76	69	58	62	60	0.90
LR	60	75	67	56	75	65	0.56

Classification was conducted with optimized balanced accuracy and performances assessed by leave-one-pair-out cross-validation using linear discriminant analysis (LDA), support vector machines based on a radial kernel (R-SVM) or linear kernel (L-SVM), random forest (RF), naïve Bayes (NB), COX risk score (COX-RS), and logistic regression (LR). ^a^ Mean of sensitivity and specificity. All measures are specified in percent. ^b^ *p*-value determined by a one-sided two-proportion z-test comparing the estimated significance between the seven machine learning methods using mRNA or lncRNA.

**Table A4:** Integrated voting results for ER positive BC patients following optimized balanced accuracy classification.

mRNA				lncRNA			
No. of Rec. Votes	Sensitivity	Specificity	Accuracy ^a^	Sensitivity	Specificity	Accuracy ^a^	*p* ^b^
≥1	85	45	65	91	36	64	0.51
≥2	84	53	68	85	56	71	0.37
≥3	75	67	71	76	62	69	0.57
≥4	65	71	68	64	73	68	0.50
≥5	60	78	69	53	80	66	0.63
≥6	56	85	71	36	85	61	0.92
7	27	95	61	22	89	55	0.78

The voting results were obtained using seven distinctive cutoffs ranging from 1–7, representing different degrees of classification agreement between the seven machine learning methods in terms of recurrence (rec.) votes. A cutoff of 1 meant that one or more votes for recurrence, placed the patient as high-risk whereas zero votes classified the patient as low-risk and similarly for the rest of the cutoffs. ^a^ Mean of sensitivity and specificity. All measures are indicated in percent. ^b^ *p*-value determined by a one-sided two-proportion z-test comparing the estimated significance between the seven machine learning methods using mRNA or lncRNA.
